# Functional Determinants of a Small Protein Controlling a Broadly Conserved Bacterial Sensor Kinase

**DOI:** 10.1128/JB.00305-20

**Published:** 2020-07-27

**Authors:** Srujana S. Yadavalli, Ted Goh, Jeffrey N. Carey, Gabriele Malengo, Sangeevan Vellappan, Bryce E. Nickels, Victor Sourjik, Mark Goulian, Jing Yuan

**Affiliations:** a Max Planck Institute for Terrestrial Microbiology, Marburg, Germany; b Department of Biology, University of Pennsylvania, Philadelphia, Pennsylvania, USA; c Department of Genetics, Rutgers University, Piscataway, New Jersey, USA; d Department of Biology, Swarthmore College, Swarthmore, Pennsylvania, USA; e Boston University School of Medicine, Boston, Massachusetts, USA; f Biochemistry and Molecular Biophysics Graduate Group, Perelman School of Medicine, University of Pennsylvania, Philadelphia, Pennsylvania, USA; g LOEWE Center for Synthetic Microbiology (SYNMIKRO), Marburg, Germany; h Molecular Biosciences Graduate Program, Rutgers University, Piscataway, New Jersey, USA; i Department of Physics, University of Pennsylvania, Philadelphia, Pennsylvania, USA; j Waksman Institute of Microbiology, Rutgers University, Piscataway, New Jersey, USA; Princeton University

**Keywords:** *Enterobacteriaceae*, cell signaling, membrane proteins, small protein, two-component regulatory systems

## Abstract

The PhoQ/PhoP two-component system plays a vital role in the regulation of Mg^2+^ homeostasis, resistance to acid and hyperosmotic stress, cationic antimicrobial peptides, and virulence in Escherichia coli, *Salmonella*, and related bacteria. Previous studies have shown that MgrB, a 47-amino-acid membrane protein that is part of the PhoQ/PhoP regulon, inhibits the histidine kinase PhoQ. MgrB is part of a negative-feedback loop modulating this two-component system that prevents hyperactivation of PhoQ and may also provide an entry point for additional input signals for the PhoQ/PhoP pathway. To explore the mechanism of action of MgrB, we analyzed the effects of point mutations, C-terminal truncations, and transmembrane (TM) region swaps on MgrB activity. In contrast to two other known membrane protein regulators of histidine kinases in E. coli, we found that the MgrB TM region is necessary for PhoQ inhibition. Our results indicate that the TM region mediates interactions with PhoQ and that W20 is a key residue for PhoQ/MgrB complex formation. Additionally, mutations of the MgrB cytosolic region suggest that the two N-terminal lysines play an important role in regulating PhoQ activity. Alanine-scanning mutagenesis of the periplasmic region of MgrB further indicated that, with the exception of a few highly conserved residues, most residues are not essential for MgrB’s function as a PhoQ inhibitor. Our results indicate that the regulatory function of the small protein MgrB depends on distinct contributions from multiple residues spread across the protein. Interestingly, the TM region also appears to interact with other noncognate histidine kinases in a bacterial two-hybrid assay, suggesting a potential route for evolving new small-protein modulators of histidine kinases.

**IMPORTANCE** One of the primary means by which bacteria adapt to their environment is through pairs of proteins consisting of a sensor and a response regulator. A small membrane protein, MgrB, impedes the activity of sensor protein PhoQ, thereby affecting the expression of PhoQ regulated virulence genes in pathogenic bacteria. However, it is unknown how such a small protein modulates the activity of PhoQ. Here, we studied the functional determinants of MgrB and identified specific amino acids critical for the protein's inhibitory function. Notably, we find that the membrane-spanning region is important for MgrB interaction with PhoQ. Additionally, this region appears to physically interact with other sensors, a property that may be important for evolving small protein regulators of sensor kinases.

## INTRODUCTION

Small proteins—proteins less than 50 amino acids long that are directly translated from small open reading frames—regulate a wide variety of cellular processes, but collectively, this class of proteins remains relatively understudied (reviewed in references 1 and 2). For example, hundreds to thousands of small open reading frames have been reported in Escherichia coli (3–5), Mycobacterium tuberculosis (6), and human microbiomes (7). A fraction of them have been confirmed with detections at the protein level (3–6), and the functions of most these proteins are unknown. In bacteria and bacteriophages, small proteins can be classified into two groups: secreted and nonsecreted (1). The former are mainly involved in communication or competition among organisms, whereas the latter are localized in the cytoplasm or cell membrane and play roles in diverse aspects of cell physiology. In E. coli, the list of small proteins that have been identified and verified continues to grow with improvements in computational and experimental techniques (3, 4, 8). Interestingly, nearly one-third have been shown or are predicted to be in the cytoplasmic membrane, suggesting many may be involved in sensing environmental cues and mediating stress responses.

Bacteria thrive in a multitude of niches, often under challenging physicochemical conditions, and have therefore evolved stress response systems to monitor the environment and modulate their physiology accordingly. Many of these stress responses are controlled by two-component signaling systems, one of the primary modes of bacterial signal transduction (9). The PhoQ/PhoP system in E. coli, *Salmonella*, and related Gram-negative bacteria is a well-studied example of a two-component system that is critical for virulence and facilitates adaptation to conditions of low Mg^2+^ or Ca^2+^, low pH, osmotic upshifts, and the presence of cationic antimicrobial peptides (10–14). In response to an input signal, the sensor histidine kinase PhoQ autophosphorylates and modulates the phosphorylation state of the response regulator PhoP, which functions as a transcription factor (10, 15). At least three small proteins are regulated by PhoP: the 31-amino-acid membrane protein MgtS is induced under magnesium limitation in E. coli and inhibits degradation of the magnesium transporter MgtA (16); the 30-amino-acid protein MgtR promotes the degradation of the virulence factor MgtC by the FtsH protease in Salmonella enterica serovar Typhimurium (17); and the 47-amino-acid protein MgrB directly inhibits PhoQ kinase activity, thereby mediating a negative-feedback loop (18, 19). All of these small proteins are membrane localized, regulate proteins expressed in the PhoP regulon, and modify cell responses to the environment.

MgrB orthologs are found in numerous *Enterobacteriaceae*, and PhoQ inhibition has been demonstrated explicitly for a few distinct genera. The physiological importance of this inhibition and the associated negative-feedback loop remain poorly understood. MgrB could serve as a point of control for additional input signals that modulate PhoQ. For example, MgrB activity is affected by the redox state of the periplasm via the protein’s two conserved periplasmic cysteines (20). MgrB is not essential and appears to mainly act in a regulatory role; however, it can have a significant impact on bacterial physiology. In E. coli, recent work has shown that strong stimulation of the PhoQ/PhoP system hinders cell division (21). Cells grown in low levels of Mg^2+^ in the absence of MgrB form filaments due to strong activation of PhoQ under these conditions. MgrB-mediated feedback inhibition thus appears to be important to appropriately limit PhoQ activity and prevent hyperactivation of the system. In at least some contexts, loss of *mgrB* can also confer a fitness advantage: mutation of *mgrB* has emerged as one of the primary mechanisms of acquired resistance to the last-resort antibiotic colistin in clinical isolates of Klebsiella pneumoniae (22–25).

In addition to MgrB, the PhoQ histidine kinase is modulated by another membrane protein, SafA (26), which is present exclusively in E. coli and acts as a connector between the EvgS/EvgA and PhoQ/PhoP two-component systems. Stimulation of EvgS by low pH upregulates SafA expression (27), which activates PhoQ kinase activity and leads to increased acid resistance. In at least some E. coli isolates, this connection is required for PhoQ activation in response to acid stress (28, 29). Another example of a connector protein that modulates the activity of a histidine kinase is MzrA (30), which links the CpxA/CpxR and EnvZ/OmpR two-component systems. Although not as small as MgrB, both SafA and MzrA are relatively small for membrane proteins (65 and 127 amino acids, respectively) and, like MgrB, are bitopic, with their N termini in the cytoplasm.

MgrB acts by inhibiting PhoQ kinase activity (19); however, the mechanism by which this small protein inhibits a protein that is roughly 10 times larger than itself and the sequence determinants responsible for this action are largely unknown. In this work, we show that the transmembrane (TM) region of MgrB is required for the protein to form a complex with its target kinase, PhoQ, in contrast to SafA and MzrA, which do not require their TM regions for their activity (31, 32). Using site-directed mutagenesis combined with MgrB functional assays and *in vivo* Förster resonance energy transfer (FRET), we determined W20 in the MgrB TM region to be crucial for MgrB/PhoQ complex formation. Additionally, we identified 11 residues across different regions of MgrB that are important for its inhibitory function. Among these residues, two lysines in the cytoplasmic region likely act through their charged side chains to regulate PhoQ HAMP conformation, although they do not appear to affect MgrB/PhoQ complex formation. Surprisingly, a large number of amino acids in the small periplasmic region of MgrB can be replaced with alanine with only minor effects on protein function. Together, our findings suggest that PhoQ inhibition by MgrB depends on coordinated interactions from amino acid residues spanning different regions of the protein. We also uncovered interactions of MgrB with other histidine kinases in bacterial two-hybrid assays, which may provide a starting point for evolving novel small membrane proteins that modulate other histidine kinases.

## RESULTS

### Sequence analysis of MgrB.

**FIG 1:**
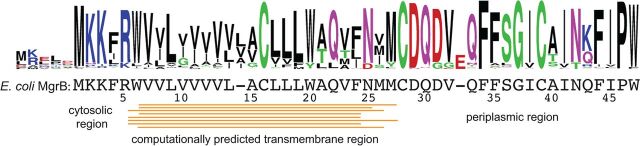
Conservation of MgrB sequence. The sequence logo of MgrB generated with bacterial MgrB sequences obtained from KEGG protein BLAST using E. coli MgrB as the query highlights the degree of amino acid conservation. The MgrB multiple-sequence alignment can be found in File S1 in the supplemental material.

MgrB was shown to be a membrane-localized small protein with N-terminal-in and C-terminal-out topology (18). To predict the TM region in MgrB, we used eight algorithms (PHDHTM, DAS-TMFILTER, SCAMPI, TMHMM, TMpred, TMMTOP, PRED-TMR, and MEMSAT) to analyze the entire amino acid sequence (Fig. 1). The predictions were fairly convergent on the TM cytoplasm-membrane boundary, which was located at W6 or V7. In contrast, the predictions of the membrane-periplasm boundary were quite different among these programs, ranging from F24 to M27. Here, we use W6 to F24 as the predicted transmembrane region of MgrB.

To gain insight into potential functionally important residues in MgrB, we aligned over 100 bacterial MgrB sequences obtained from KEGG protein BLAST, using E. coli MgrB as the query (Fig. 1; see File S1 in the supplemental material). As indicated in the sequence logo of the alignment, the amino acids were fairly conserved in the cytosolic and periplasmic regions. This high degree of conservation might originate from limitations in the diversity of MgrB sequences currently available in databases or suggest the functional importance of each conserved amino acid in these regions. In contrast, more variations and insertion of one amino acid were shown in the predicted TM region. Interestingly, two amino acids with polar side chains (C16 and Q22) and two tryptophans (W6 and W20) in the TM region are highly conserved, suggesting functional importance.

### MgrB TM and periplasmic regions are necessary but not sufficient for MgrB function.

**FIG 2:**
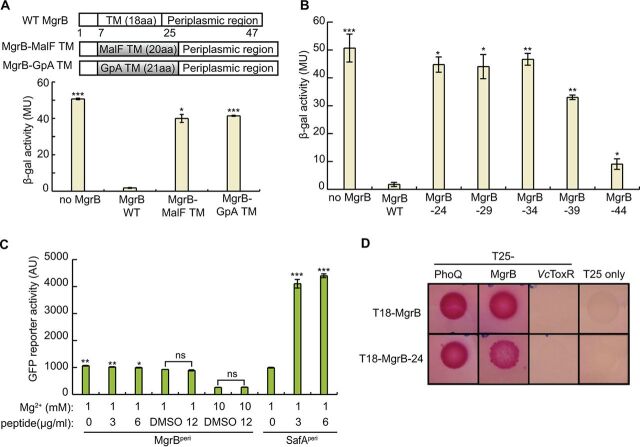
The MgrB transmembrane domain and periplasmic domain are essential but not sufficient for its function as a PhoQ inhibitor. (A) Schematic representation of TM domain swap constructs to replace the MgrB TM sequence with TM sequences derived from either the protein MalF or human GpA. β-Galactosidase activities of wild-type MgrB (pAL38), its variants MgrB-MalF TM (pSY3) and MgrB-GpA TM (pSY4), a and no-MgrB control (pAL39) in a *ΔmgrB* strain containing a PhoQ/PhoP-regulated transcriptional reporter, P*_mgtA_-lacZ* (AML67), are shown below. Inducer was not added, as leaky expression from the *trc* promoter resulted in sufficient levels for strong inhibition by WT MgrB. The data represent averages and ranges for two independent experiments. MU, Miller units; aa, amino acids. (B) Effects of wild-type MgrB and the truncation mutants were measured in a *ΔmgrB* strain containing the PhoQ/PhoP-regulated transcriptional reporter P*_mgtA_-lacZ* (AML67) as described for panel A. (C) The activities of MgrB^Peri^ and SafA^Peri^ were monitored using the PhoQ/PhoP-regulated transcriptional reporter P*_mgtLA_-gfp*. Peptides or DMSO was added to the E. coli *imp-4213* culture in the indicated amounts. The GFP activities were measured after 2 h of incubation using flow cytometry. The data points represent averages of the results of three independent experiments, and the error bars show standard deviations (SD). (D) BACTH colorimetric spot assays with cells expressing protein fusions to the T18 and T25 subunits of Bordetella pertussis adenylyl cyclase. Representative images show interactions between MgrB-24 (T18–MgrB-24) and partner proteins PhoQ (T25-PhoQ) and MgrB (T25-MgrB). Interactions between MgrB-PhoQ, MgrB-MgrB, MgrB-T25 only, and (MgrB-24)-T25 only were included as positive and negative controls. As an additional control, interactions between MgrB-24 and the V. cholerae membrane protein ToxR (*Vc*ToxR), and also between MgrB and *Vc*ToxR, were tested. Each pair of interactions was assayed in at least three independent trials in a *cyaA* mutant BACTH host strain containing *lacI*^q^ (SAM85) on maltose-MacConkey indicator plates (results from one representative experiment are shown). The plates were incubated at 30°C for 48 h before images of the plates were acquired. The pink color indicates reconstitution of adenylyl cyclase activity. (A to C) The statistical significances of changes in reporter activity were calculated with *t* tests by comparison to the wild type (A and B) or to the DMSO negative control (C), and significance is indicated with asterisks (***, *P ≤ *0.001; **, *P ≤ *0.01; *, *P ≤ *0.05; ns, not significant).

As a first step toward understanding how MgrB inhibits PhoQ at a structural level, we looked at the role of the protein’s TM and periplasmic regions. To test the functional role of the MgrB TM region, we swapped the predicted MgrB TM region with either (i) a 20-amino-acid TM from the E. coli maltose transporter MalF or (ii) a 21-amino-acid variant of the human glycophorin A (GpA) TM (33) (Fig. 2A). The MgrB variants described here are derivatives of a functional N-terminally green fluorescent protein (GFP)-tagged MgrB (GFP-MgrB) (18). Both MgrB variants with swapped TM regions localized to the cell membrane (see Fig. S1 in the supplemental material), and their expression levels were comparable to that of the wild type (WT) (see Fig. S2 in the supplemental material). TM-swapping experiments with two previously studied histidine kinase regulators—SafA and MzrA—indicated that the transmembrane sequence was dispensable for their functions (31, 32). Indeed, our control experiments, in which we replaced the TM sequences of SafA and MzrA with that of GpA, confirmed these results (see Fig. S3 in the supplemental material). Thus, for SafA and MzrA, the TM region appears to act primarily as an anchor to direct the regulatory domains of the proteins to the membrane. We monitored the effects of MgrB and its TM swap variants on PhoQ activity using a PhoQ/PhoP-regulated transcriptional reporter (P*_mgtA_-lacZ*) (Fig. 2A). Control cells expressing no MgrB had a high level of β-galactosidase (β-Gal) activity, indicating uninhibited PhoQ, whereas cells expressing WT MgrB had minimal β-Gal activity, indicating PhoQ inhibition by WT MgrB, as expected. In contrast to the inhibitory function of the WT MgrB, both MgrB TM swap variants (MgrB-MalF TM and MgrB-GpA TM) showed high β-Gal activities that were about 80% of the activity of the no-MgrB control, indicating that they were unable to inhibit PhoQ efficiently. These results suggest that the MgrB TM region not only functions as a membrane anchor but also has additional properties that are critical for MgrB to inhibit PhoQ.

As swapping the TM sequence in MgrB severely affected its activity, we tested whether an MgrB mutant lacking the periplasmic region inhibits PhoQ. This variant—MgrB-24, in which N25 is replaced with a stop codon—showed proper membrane localization (see Fig. S1) but could not repress reporter gene expression (Fig. 2B), indicating that this truncated variant composed of the MgrB TM and short N-terminal cytoplasmic regions is not functional. To assess the minimum length of MgrB needed for efficient repression of PhoQ/PhoP-regulated reporter gene expression, we tested other C-terminal truncations of MgrB: MgrB-29, MgrB-34, MgrB-39, and MgrB-44, with replacement of the codons for Q30, F35, A40, and I45 with a stop codon, respectively. These mutants displayed membrane localization similar to that of WT MgrB (see Fig. S1). Of the four truncations, MgrB-29 and -34 did not show any detectable repression; MgrB-39 showed slight repression; and MgrB-44, which lacks only the last two residues, showed the strongest repression, although the variant was still compromised in activity compared with the WT (Fig. 2B). Evidently, amino acid interactions in both the TM and the periplasmic regions of MgrB influence the protein’s ability to inhibit PhoQ.

Previous work suggested that the periplasmic region of MgrB is required for PhoQ inhibition (18). Since the TM swaps described above change the TM helix length, their loss of activity could be due to a change in the helical phase at the C terminus of the TM helix, creating different orientations of the periplasmic region. It is therefore possible that the periplasmic region of MgrB alone could inhibit PhoQ, while the TM helix only ensures its correct orientation. Indeed, the chemically synthesized periplasmic region of SafA (SafA^peri^) was previously shown to be sufficient for PhoQ activation when added exogenously (34). Therefore, we tested whether the MgrB periplasmic region (MgrB^peri^) could function similarly to full-length MgrB (Fig. 2C) by adding MgrB^peri^ to an E. coli culture. To enable the MgrB^peri^ peptide to access the periplasm, we used an *imp-4213* strain, which has increased outer membrane permeability (35). We monitored PhoQ activity with a GFP reporter fused to the promoter region of a PhoQ/PhoP-regulated gene (pUA66 P*_mgtLA_-gfp*) in the presence or absence of chemically synthesized MgrB^peri^. The positive control, chemically synthesized SafA^peri^, enhanced PhoQ activity significantly, consistent with a previous report (34). In contrast, MgrB^peri^ did not show any inhibitory effect on PhoQ activity in the concentration range that we tested, though dimethyl sulfoxide (DMSO), the solvent for MgrB^peri^ peptide, showed a slight inhibition of *gfp* reporter expression. As MgrB^peri^ is short and likely capable of sampling different conformations, our results suggest that, unlike SafA, the MgrB periplasmic region alone is not sufficient to inhibit PhoQ. Taken together, the above-described results indicate that both the MgrB TM and periplasmic regions are required but not sufficient to inhibit PhoQ.

### MgrB lacking its periplasmic region is capable of physical interaction with PhoQ.

Although MgrB-24 (the N-terminal region plus the TM region) does not inhibit PhoQ, it could still play a role in establishing physical contact with PhoQ. To explore this possibility, we used a bacterial two-hybrid (BACTH) system based on split adenylyl cyclase (36, 37). In the assay, reconstitution of the T18 and T25 fragments restores the activity of adenylate cyclase (CyaA), which leads to an increase in cAMP levels. This increased cAMP, in turn, enables efficient induction of the genes required for maltose catabolism, resulting in pink colonies on maltose MacConkey agar plates. To assay interactions of MgrB lacking its periplasmic region with PhoQ, we prepared N-terminal fusions of MgrB-24 and PhoQ to T18 and T25 fragments, respectively. As controls, we tested interactions between MgrB-PhoQ and MgrB-*Vc*ToxR (the Vibrio cholerae inner membrane protein ToxR), as well as the MgrB-T25 fragment alone. Spot assays of cells expressing T18–MgrB-24 and T25-PhoQ produced a dark-pink color after 48 h at 30°C, whereas cells expressing T18–MgrB-24 and the T25 fragment alone were colorless (Fig. 2D). Spots of cells expressing T18–MgrB-24 and either T25-MgrB or T25–MgrB-24 were also pink. Control cells expressing T18-MgrB and either T25-PhoQ or T25-MgrB formed dark-pink spots, showing an interaction of MgrB with PhoQ and possibly with itself, consistent with previous work (18), whereas control cells expressing either T18-MgrB or T18–MgrB-24 with either T25-*Vc*ToxR or the T25 fragment alone remained colorless. Taken together, these results suggest that the MgrB N terminus-plus-TM region interacts with PhoQ and may also interact with itself to facilitate formation of a multimeric complex.

### A small number of functionally important residues are spread across different regions of MgrB.

To explore the relative importance of different residues in MgrB, we made alanine substitutions and analyzed their effects on PhoQ inhibition. Interestingly, a majority of the substitutions in MgrB did not affect PhoQ/PhoP reporter gene expression, but 11 substitutions spread across different regions showed significant activation of the reporter gene (see Fig. 5; see Fig. S4 and S5 in the supplemental material), indicating impaired MgrB activity. We confirmed that each of the 11 MgrB variants was localized to the membrane with expression levels that were comparable to that of wild-type MgrB (see Fig. S1 and S2). Below, we discuss these 11 MgrB variants in more detail.

**FIG 3:**
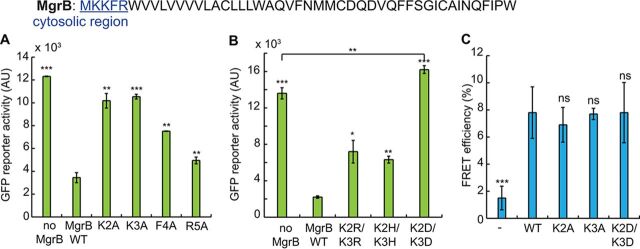
Two lysines in the MgrB cytosolic region regulate PhoQ activity through their charged side chain. (A) The activities of MgrB mutants with single alanine substitutions in the cytosolic region were tested using the PhoQ/PhoP-regulated transcriptional reporter P*_mgtLA_-gfp* in a *ΔmgrB* strain. MgrB mutants were constructed in a pBAD33 vector, and their expression was induced with 0.008% arabinose. (B) The activities of MgrB K2 K3 double mutants were tested as for panel A. The reporter activity change was significant (*P = *0.0045) when comparing MgrB K2D K3D to the no-MgrB control. (C) FRET efficiencies between PhoQ-mNeonGreen and mCherry (−), mCherry-tagged wild-type MgrB (WT), or MgrB mutants (K2A, K3A, K2D K3D, and K2E K3E) were measured. The MG1655 *ΔphoQ ΔmgrB* strain was transformed with pBAD33 *phoQ-mneongreen* and pTrc99a *mcherry-mgrB* variants. Protein expression was induced with 0.008% arabinose and 10 μM IPTG. FRET was measured for each protein pair, and the efficiencies were calculated as described in Materials and Methods. All the data points represent averages of the results of at least three independent experiments, and the error bars show SD. The statistical significances of changes in reporter activity or FRET efficiency were calculated with *t* tests by comparison to the wild type or as specified and are indicated with asterisks (***, *P ≤ *0.001; **, *P ≤ *0.01; *, *P ≤ *0.05; ns, not significant).

**(i) Two lysines in the MgrB cytosolic region regulate PhoQ activity.** In the short N-terminal cytosolic region of MgrB (M1 to R5), we made alanine substitutions at each position except the first methionine. Here, we used the PhoQ/PhoP-regulated GFP reporter (pUA66 P*_mgtLA_-gfp*) to monitor the activities of MgrB variants. Control cells having no MgrB expression showed a high intensity of GFP fluorescence, while cells expressing wild-type MgrB had much lower reporter fluorescence (Fig. 3A). The K2A and K3A substitutions displayed the most effect on *gfp* reporter expression, with fluorescence comparable to that of the no-MgrB control (Fig. 3A). F4A and R5A had about 50% and 80% activity, respectively. K2A and K3A, however, retained only about 20% activity (Fig. 3A) (see Materials and Methods for the definition of normalized activity).

To further investigate whether this loss of activity might be caused by deficient protein interactions, we used *in vivo* acceptor photobleaching FRET (38) to test complex formation between the two MgrB variants and PhoQ. In this assay, the MgrB variants and PhoQ were tagged with a red fluorescent protein (mCherry) and a green fluorescent protein (mNeonGreen), respectively, and coexpressed in an E. coli *ΔphoQ ΔmgrB* strain. Complex formation between tagged proteins typically results in close proximity and consequently energy transfer between the donor mNeonGreen and the acceptor mCherry, leading to quenching of the mNeonGreen fluorescence. The latter can be detected as an increase in the mNeonGreen fluorescence upon photobleaching of mCherry that abolishes FRET (see Materials and Methods). The apparent FRET efficiency could be defined as the resulting increase in the donor fluorescence divided by its fluorescence after bleaching (39). While apparent FRET efficiencies may vary depending on the optical setup, for intramolecular mNeonGreen-mCherry tandem constructs, the reported values are in the range of 15% to 20% (40). We observed about 7.0% FRET efficiency for PhoQ-mNeonGreen/mCherry-MgrB WT, whereas the negative control, PhoQ-mNeonGreen/mCherry, showed only about 1.5% FRET efficiency. Interestingly, K2A and K3A mutants showed FRET efficiencies similar to that of MgrB WT (Fig. 3C), suggesting that substitutions at these two positions do not significantly affect MgrB/PhoQ complex formation. Therefore, K2 and K3 may instead be directly involved in regulating PhoQ conformation and promoting a kinase-inactive state of the enzyme.

We speculated that MgrB might regulate PhoQ conformation through the positively charged side chains of K2 and K3. To test this hypothesis, we mutated K2 or K3 to a negatively charged amino acid. Mutating K2 or K3 individually to aspartate only slightly reduced MgrB function (see Fig. S4A and B). Furthermore, when we replaced these two lysines individually with 19 other amino acids, many of the mutants retained more than 50% activity (see Fig. S4A and B). Thus, neither K2 nor K3 alone is essential for MgrB function. We then tested the effect of replacing both K2 and K3 with aspartate. MgrB K2D K3D localized to the cell membrane similarly to wild-type MgrB (see Fig. S1) but with some reduced expression (see Fig. S2). At this expression level, the K2D K3D mutant appeared to be a mild PhoQ activator rather than an inhibitor and showed 20% higher reporter activity than the no-MgrB control (Fig. 3B). Increasing MgrB K2D K3D expression, either by doubling the amount of arabinose inducer or by using a stronger promoter (the *trc* promoter), produced a low level of inhibition that was much less than the level of inhibition of wild-type MgrB (see Fig. S4C and D). Double mutants, in which the lysines were replaced with either the positively charged amino acid histidine or arginine (K2H K3H and K2R K3R), on the other hand, retained about 60% inhibitory activity (Fig. 3B). FRET analyses also showed that MgrB K2D K3D had a FRET efficiency comparable to that of the wild type (Fig. 3C), indicating that this MgrB variant forms complexes with PhoQ. Taken together, our results suggest that the two lysines in the MgrB cytosolic region regulate PhoQ function through their positively charged side chains without contributing to PhoQ/MgrB complex formation in E. coli. We interpret the fact that single amino acid replacements of K2 or K3 with uncharged residues has little effect on activity as indicating that the remaining lysine is sufficient to mediate the required interaction. We also note that the K3R substitution was inactive (see Fig. S4B), which suggests that it is not only the charge of the lysine side chains that is important for MgrB activity.

**FIG 4:**
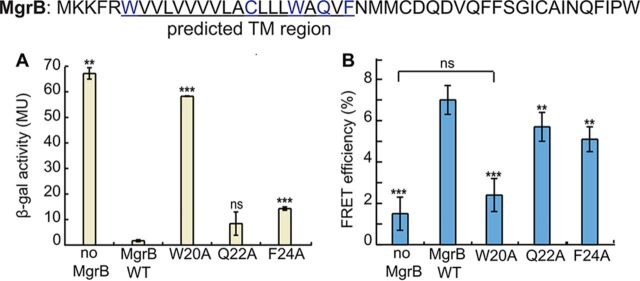
W20 in the MgrB transmembrane domain is essential for PhoQ/MgrB complex formation. (A) β-Galactosidase levels were measured in a *ΔmgrB* strain containing the PhoQ/PhoP-regulated transcriptional reporter P*_mgtA_-lacZ* (AML67) and also containing either an empty vector (pAL39), wild-type MgrB (pAL38), or the indicated MgrB W20A (pSY72), Q22A (pSY73), and F24A (pSY74) mutants. Inducer (IPTG) was not added, as leaky expression from the *trc* promoter resulted in sufficient levels of protein. Mutants with β-galactosidase activity ≥5-fold above wild type were selected for further analysis. The data represent averages and ranges for the results of two independent experiments. MU, Miller units. (B) FRET efficiencies were measured between PhoQ-mNeonGreen and the indicated mCherry-MgrB variants as described in the legend to Fig. 3C. Compared to the wild type, the reduction of FRET efficiencies was significant, according to *t* tests. The data points represent averages of the results of at least three independent experiments, and the error bars show SD. The statistical significances of changes in reporter activity or FRET efficiency were calculated with *t* tests by comparison to the wild type and are indicated with asterisks (***, *P ≤ *0.001; **, *P ≤ *0.01; ns, not significant).

**(ii) The TM helix is involved in PhoQ/MgrB complex formation through W20.** Given that the MgrB TM sequence is necessary for its function, we wanted to further identify specific residues that might contribute to PhoQ inhibition. Polar amino acids, such as N and Q, can facilitate TM domain interactions by forming stable hydrogen bonds (41). Aromatic residues are known to preferentially occur in lipid-water interface regions (42, 43) and could be important for membrane anchoring, as well as surface interactions. An MgrB sequence alignment identified four polar or aromatic residues in the transmembrane region that are highly conserved: W6, C16, W20, and Q22. Based on these considerations, we mutated the four highly conserved residues and F24 individually to alanine. The W6A and C16A substitutions did not affect MgrB function (Fig. 4A; see Fig. S5); the result for C16A is consistent with previous observations (20). The F24A substitutions displayed modest effects on reporter gene expression (∼8-fold higher β-Gal activity than for WT MgrB), and the change in activity for the Q22A mutant was not statistically significant (Fig. 4A). The W20A substitution, on the other hand, greatly affected MgrB function, with β-Gal reporter activity that was ∼30-fold higher than that of WT MgrB (Fig. 4A). These results were also reproduced with the GFP reporter system and untagged MgrB (see Fig. S5), except that the Q22A substitution showed significant activity loss (∼70%) in this case. Collectively, the results from both approaches indicate that W20, F24, and possibly Q22 in the TM region are functionally important.

Our bacterial two-hybrid data suggest that the MgrB TM region plays a role in establishing physical contact with PhoQ. To pinpoint the essential residues in the MgrB TM region that are responsible for MgrB/PhoQ complex formation, we performed FRET analyses on MgrB TM point mutants (Fig. 4B). Compared to wild-type MgrB, which had 7.0% FRET efficiency, Q22A and F24A showed slightly reduced FRET efficiency: 5.7% and 5.2%, respectively. W20A, on the other hand, produced a much greater change, with FRET efficiency reduced to 2.4%, which was comparable to that of the negative control. These results indicate that W20 in the TM region is a key residue for MgrB/PhoQ complex formation.

**FIG 5:**
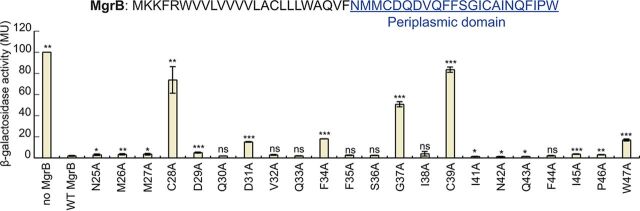
Effects of site-directed alanine substitutions of MgrB periplasmic residues on PhoQ activity. β-Galactosidase levels were measured in a *ΔmgrB* strain containing the PhoQ/PhoP-regulated transcriptional reporter P*_mgtA_-lacZ* (AML67) and also containing either an empty vector (pAL39), wild-type MgrB (pAL38), or the indicated mutants (pJC1 through pJC7 and pTG1 through pTG15) (see Table S1B for details). Inducer (IPTG) was not added, as leaky expression from the *trc* promoter resulted in sufficient levels of protein. Data for wild-type MgrB and each mutant were normalized to the no-MgrB control. Mutants with β-galactosidase activity ≥5-fold above wild type were selected for further analysis. Averages and standard deviations for the results of three independent experiments are shown. MU, Miller units. The statistical significances of changes in reporter activity were calculated with *t* tests by comparison to the wild type and are indicated with asterisks (***, *P ≤ *0.001; **, *P ≤ *0.01; *, *P ≤ *0.05; ns, not significant).

**(iii) The periplasmic region has six functionally important residues.** In the periplasmic region, mutants C28A and C39A showed the greatest effects (80- to 90-fold higher β-Gal activity than WT MgrB), consistent with previous data indicating that these cysteines are important for MgrB activity (20). In addition, the G37A, D31A, F34A, and W47A mutants displayed significant increases in reporter gene expression—approximately 30-, 8-, 10-, and 10-fold relative to WT MgrB, respectively (Fig. 5). These results were also reproduced with the GFP reporter system and untagged MgrB constructs (see Fig. S5). The fact that mutating residues C28, G37, and C39 showed the strongest effect on reporter activity suggests that these residues are likely important for structural stability of the MgrB periplasmic region.

### A charged residue in the PhoQ TM2-HAMP junction is involved in MgrB inhibition.

**FIG 6:**
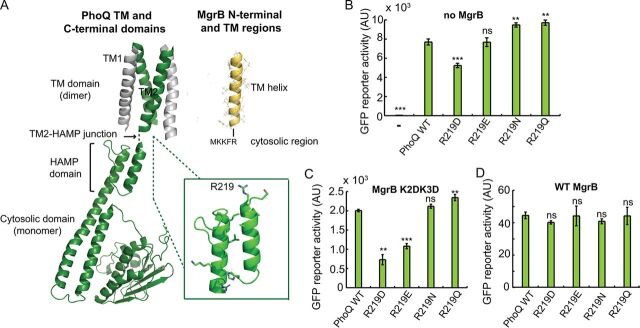
PhoQ R219 is involved in interactions with MgrB K2 and K3. (A) Models of TM (14) and cytosolic (47) domains of PhoQ and MgrB. The MgrB TM model was constructed *ab initio* with Rosetta (https://www.rosettacommons.org). The PhoQ R219 residue is labeled and shown with its side chain in an enlarged view. (B) The activities of PhoQ R219 mutants were tested using the PhoQ/PhoP-regulated transcriptional reporter P*_mgtLA_-gfp* in a *ΔphoQ ΔmgrB* strain. PhoQ mutants were expressed from the plasmid pBAD33 with 0.008% arabinose. (C and D) The activities of PhoQ R219 mutants were tested in the presence of MgrB K2D K3D (C) or the wild type (D) using the same GFP reporter as for panel B. The MgrB variants were expressed from a pTrc99a vector with no inducer (IPTG) added, as leaky expression from the *trc* promoter resulted in sufficient levels of protein. (B to D) Averages and standard deviations for three independent experiments are shown. The statistical significances of changes in reporter activity were calculated with *t* tests by comparison to the wild type and are indicated with asterisks (***, *P ≤ *0.001; **, *P ≤ *0.01; ns, not significant). AU, arbitrary units.

Our results suggest that the two N-terminal lysines in MgrB regulate PhoQ activity through their charged side chains. To further explore which PhoQ residue(s) in PhoQ interacts with the lysines, we screened for PhoQ mutants for which MgrB K2D K3D showed increased inhibitory activity. Based on the MgrB and PhoQ topologies, we hypothesized that K2 and K3 of MgrB interact with residues in the PhoQ TM2-HAMP region (Fig. 6A). We therefore mutated this region of PhoQ by error-prone PCR and screened for PhoQ mutants that had reduced activity in the presence of MgrB K2D K3D. About 100 colonies with decreased green fluorescent reporter protein were selected, and the *phoQ* genes were sequenced. Mutants with missense mutations are highlighted in File S2 in the supplemental material. Most of the missense mutations were clustered in/near the TM2-HAMP junction and the second helix of the HAMP domain. We focused on the former, as residues in this region are more likely to be close enough to interact with the lysines in MgrB. Among them, R219 (labeled in Fig. 6A), which was the most frequent among identified mutations, appeared to be the most promising residue.

We therefore mutated PhoQ R219 to negatively charged amino acids (D and E), as well as amino acids with amide side chains (N and Q). Compared to wild-type PhoQ, R219D had reduced activity (about 70% of WT), while the other three mutants (R219E, R219N, and R219Q) had activities that were either similar to or slightly greater than that of the wild type (Fig. 6B). Next, we tested the activities of these PhoQ mutants in the presence of MgrB K2D K3D. Compared to wild-type PhoQ, R219D and R219E were indeed repressed further by MgrB K2D K3D (Fig. 6C), suggesting that reversing the side chain charge of PhoQ R219 compensates for the effect of the charge inversion of MgrB N-terminal lysines. The inhibition of PhoQ R219N and R219Q by MgrB K2D K3D, on the other hand, was comparable to the inhibition of wild-type PhoQ, indicating that charge, but not hydrogen bonding, is important for interactions of R219D and R219E with MgrB K2D K3D. We also found that wild-type MgrB inhibited all four PhoQ R219 mutants to an extent similar to the inhibition of wild-type PhoQ (Fig. 6D), suggesting the existence of other factors involved in PhoQ inhibition by wild-type MgrB.

### Inhibition of E. coli PhoQ by MgrB orthologs.

**FIG 7:**
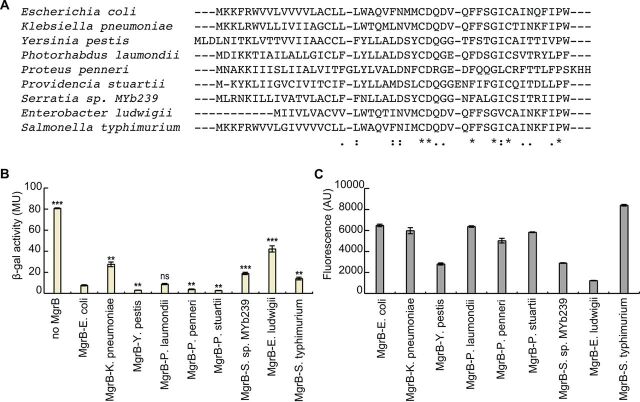
Effects of MgrB orthologs on E. coli PhoQ activity. (A) Sequence alignment of MgrB orthologs (*, fully conserved residues; :, conservation between strongly similar residues; ., conservation between weakly similar residues) . (B) β-Galactosidase levels of a *ΔmgrB* strain containing the PhoQ/PhoP-regulated transcriptional reporter P*_mgtA_-lacZ* (AML67) and also containing either an empty vector (pAL39), wild-type MgrB (pAL38), or a plasmid expressing the indicated MgrB orthologs. Inducer (IPTG) was not added, as leaky expression from the *trc* promoter resulted in sufficient levels of protein. Averages and standard deviations for the results of three independent experiments are shown. MU, Miller units. The statistical significances of changes in reporter activity were calculated with *t* tests by comparison to the wild type and are indicated with asterisks (***, *P ≤ *0.001; **, *P ≤ *0.01; ns, not significant). (C) GFP-MgrB expression for cells expressing MgrB orthologs was imaged by fluorescence microscopy, and GFP fluorescence levels were quantified (see Materials and Methods for details). The data represent averages and ranges for the results of two independent experiments comprised of 200 cells each, AU, arbitrary units.

MgrB orthologs have previously been identified in several enterobacterial species (18). To examine the activities of these MgrB natural variants on E. coli PhoQ, we expressed MgrB from K. pneumoniae, Yesinia pestis, Photorhabdus laumondii, Proteus penneri, Providencia stuartii, *Serratia* sp. strain MYb239, Enterobacter ludwigii, and S. enterica serovar Typhimurium in E. coli K-12 MG1655. All the orthologs except one (E. ludwigii MgrB) localized to the membrane (see Fig. S1F). E. ludwigii MgrB, which is missing the positively charged residues (K2 and K3) in its N terminus, showed defective localization, suggesting the lack of a signal peptide. We found that most of the orthologs were expressed at levels comparable to that of E. coli MgrB, but the ortholog from E. ludwigii was expressed quite poorly in E. coli (∼5-fold lower than E. coli MgrB) (Fig. 7). We then measured reporter gene expression for PhoP-regulated transcription as a function of the activity of each MgrB ortholog (Fig. 7). Unsurprisingly, E. ludwigii showed high reporter gene expression, indicating low PhoQ inhibition. MgrB from K. pneumoniae, *Serratia* sp. MYb239, and *S.* Typhimurium showed small but significant increases in reporter activity (∼4-fold, 2.5-fold, and 2-fold, respectively). Interestingly, Y. pestis, P. penneri, and P. stuartii MgrB proteins produced reporter activities that were significantly lower than that of E. coli MgrB, suggesting that they repress E. coli PhoQ more strongly than does E. coli MgrB.

### MgrB interaction with noncognate histidine kinases.

**FIG 8:**
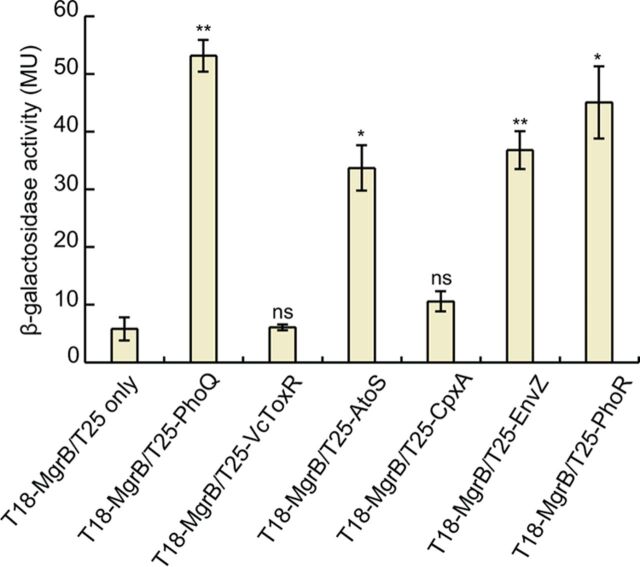
MgrB interacts with other histidine kinases in a BACTH assay. BACTH assays with cells expressing protein fusions to the T18 and T25 subunits of B. pertussis adenylyl cyclase were performed. β-Gal expression from the *lac* promoter was monitored as a measure of interactions between T18-MgrB and a T25 fusion with either PhoQ, AtoS, CpxA, EnvZ, or PhoR. The T25 fragment alone and T25-*Vc*ToxR served as negative controls. The β-Gal assays were carried out in a *cyaA* mutant BACTH host strain containing *lacI*^q^ (SAM85). The data represent averages and ranges for two independent experiments. MU, Miller units. The statistical significances of changes in reporter activity were calculated with *t* tests by comparison to the negative control (T18-MgrB/T25 only) and are indicated with asterisks (**, *P ≤ *0.01; *, *P ≤ *0.05; ns, not significant).

To test if MgrB is capable of physically interacting with histidine kinases other than PhoQ, we made constructs of E. coli EnvZ, AtoS, CpxA, and PhoR fused to the T25 fragment of adenylyl cyclase and performed BACTH analysis by spot assays, as well as by measuring β-Gal activities of liquid cultures. Intriguingly, cells expressing T18-MgrB and either T25-AtoS, -EnvZ, or -PhoR showed a high level of activity, although less than the β-Gal activity of cells expressing T18-MgrB and T25-PhoQ (Fig. 8). Cells expressing T18-MgrB and T25-CpxA, on the other hand, displayed a level of β-Gal activity only marginally higher than that of the negative controls. These results suggest that MgrB is capable of physically interacting with EnvZ, AtoS, and PhoR, at least in the context of the bacterial two-hybrid assay. To test whether MgrB affects the activity of these histidine kinases, we measured gene expression from promoters regulated by the histidine kinases in the presence or absence of plasmid-driven MgrB expression. However, we did not see any effect of MgrB on gene expression controlled by any of the histidine kinases (data not shown). We also tested several other histidine kinases: ArcB, BaeS, BasS, CpxA, CusS, EvgS, NtrB, RstB, and TorS (see Fig. S6 in the supplemental material). None of them showed interactions with MgrB that were comparable to that of PhoQ.

## DISCUSSION

In this study, we explored the regions of the small protein MgrB that are important for its activity as an inhibitor of the PhoQ sensor kinase. Using a combination of biochemical and biophysical approaches, we identified 11 amino acids in MgrB that, at least in the context of alanine substitution, are important for the protein’s inhibition of PhoQ. These functionally important residues are spread across different regions of the molecule, consistent with our observation that TM replacement or periplasmic-region truncation leads to a loss of MgrB function. The fact that the MgrB TM sequence is required for the protein’s activity distinguishes MgrB from at least two other bitopic membrane proteins that regulate histidine kinases (SafA and MzrA); the TM domains of SafA and MzrA apparently serve primarily as membrane anchors (31, 32). In addition, our results indicate that the MgrB TM region, together with the small cytoplasmic region, interacts with PhoQ and that residue W20 in the TM region is essential for MgrB/PhoQ complex formation. Tryptophan is often found in transmembrane proteins, especially at the ends of TM helixes as a membrane anchor due to its unique amphipathic side chain (44, 45). W20 in MgrB does not appear to be positioned near the computationally predicted membrane-periplasm interface. It is possible that the unique side chain of W20 interacts with polar and/or aromatic residues in the PhoQ transmembrane domain, such as the functionally important N202 (46). The mechanism by which W20 mediates PhoQ/MgrB complex formation awaits further investigation.

We identified two lysines in the MgrB cytosolic region that are important for the protein’s function. Although these residues are not individually essential, replacing both with negatively charged residues significantly affected MgrB activity. A screen for PhoQ mutants that increase the inhibitory activity of MgrB K2D K3D identified R219 in PhoQ. When this residue was mutated to a negatively charged amino acid, the inhibitory activity of MgrB K2D K3D was partially restored, suggesting the possibility that the MgrB-inhibitory activity of PhoQ is in part mediated by charge-charge repulsion between K2 K3 in MgrB and R219 in the PhoQ TM2-HAMP junction. This hypothesis is consistent with the observation that K2 and K3 did not contribute to MgrB/PhoQ complex formation, since the HAMP domain is conformationally dynamic and transduces signals via conformational changes (47, 48). It is also noteworthy that the TM2-HAMP junction has been reported to be essential for signaling by other membrane proteins, such as the chemoreceptor Tar (49). In fact, some modifications of the TM2-HAMP junction result in signal inversion in E. coli (49). The partial suppression by PhoQ R219D indicates the existence of other factors involved in PhoQ/MgrB interaction at this residue. Mutating arginine to aspartate changes not only the charge but also the length of the side chain (as well as other properties), which may have an impact on PhoQ/MgrB interaction. Interestingly, we observed activity inversion (from activation to inhibition) of MgrB K2D K3D when increasing its expression. Considering its reduced membrane localization and possible heterogeneous cell population under arabinose induction, it is possible that the mild activating activity we observed for a lower expression level was due to factors other than the direct effects of the mutant MgrB on PhoQ. Alternatively, different amounts of MgrB K2D K3D might lead to different energy states or conformations of the PhoQ TM2-HAMP junction and thus changes in PhoQ activity. Lastly, we note that some MgrB orthologs have a longer N-terminal cytosolic region and some do not have positively charged residues at positions 2 and 3. Due to the dynamic nature of the HAMP domain and the TM2-HAMP junction, it is possible that these MgrB orthologs have different interactions with PhoQ.

Previous work indicated that the periplasmic region of MgrB and, in particular, two conserved cysteines in the region are critical for PhoQ inhibition (18, 20). To identify other periplasmic residues in MgrB that are required for PhoQ inhibition, we analyzed individual alanine substitutions across the entire periplasmic region. Remarkably, despite the small size of the region and the level of conservation, substitutions at most positions had little effect on MgrB activity. Of the 22 residues in the periplasmic region, only 6 were individually important for full activity: the two conserved cysteines (C28 and C39), as previously noted (20), as well as G37, D31, F34, and W47. Furthermore, substitutions at the last four residues lowered but did not eliminate MgrB activity. Interestingly, mutations at each of the positions are also associated with colistin-resistant *Klebsiella* isolates (23, 24, 50–55). Taken together, our findings show that the sequence determinants for MgrB activity are not restricted to any one of the regions—cytosolic, periplasmic, or TM—and that functionality is mediated by collective interactions of key amino acid residues scattered across the polypeptide.

We also tested a number of MgrB orthologs derived from other enterobacteria with varying sequence similarity to E. coli MgrB. Most orthologs that we tested efficiently inhibited E. coli PhoQ. The slight decrease in the activities of K. pneumoniae and *S.* Typhimurium MgrB proteins could be due to the presence of a glycine residue within the TM regions, which might affect the conformational flexibility of the TM helix and cause a defect in binding and inhibiting PhoQ. On the other hand, Y. pestis, P. penneri, and P. stuartii MgrB proteins appear to be more efficient than E. coli MgrB in inhibiting E. coli PhoQ. It is notable that the tryptophan residue (W20) found in the TM region of E. coli MgrB is replaced by a tyrosine (Y20) in these orthologs. It is possible that a hydrogen bond involving Y20 or the smaller size of tyrosine relative to tryptophan affects the interaction of MgrB with PhoQ.

Unexpectedly, we also found that MgrB lacking its periplasmic region, as well as full-length MgrB, interacted with several other histidine kinases in addition to PhoQ in a bacterial two-hybrid assay. However, we did not observe a significant effect of MgrB on gene expression regulated by these histidine kinases. The TM domains of many dimeric histidine kinases, such as PhoQ and EnvZ, are believed to form a four-helix bundle (48). Our results may reflect an interaction between the MgrB TM and a conserved conformation of the transmembrane domain shared by multiple histidine kinases. The cytosolic and periplasmic regions of MgrB apparently provide additional interactions required for specific regulation of PhoQ. It will be interesting to determine if there are other small membrane proteins that have evolved to interact with other histidine kinases using structural features similar to those of the MgrB TM region. In addition, the results from our study of MgrB, as well as studies of other small membrane proteins, may provide a platform for reengineering these proteins to regulate new targets through rational design and directed evolution.

## MATERIALS AND METHODS

### Strains, plasmids, and cloning.

Information on the strains (see Table S1A in the supplemental material), plasmids (see Table S1B), and primers (see Table S1C) used in this study is provided in the supplemental material. All the strains were derived from E. coli K-12 MG1655. Gene deletions and reporter constructs were transferred between strains using transduction with P1_vir_. Deletions in the Keio collection (56) that were used for strain construction were confirmed by PCR using primers flanking the targeted gene. Kanamycin resistance markers were excised when required, using the FLP recombinase-expressing plasmid pCP20 (57). Strain SAM72 was constructed by first excising the kanamycin resistance cassette from the *ompC-mcherry* transcriptional reporter strain AFS256 to give SAM71. Kanamycin resistance from the *mzrA* deletion strain JW3067 was then transduced into SAM71 by P1 transduction. A deletion of *safA* (SAM73) was constructed as described previously (28, 57). SAM73 was then used to construct SAM76 by transducing the *mgtA-lacZ* transcriptional reporter from TIM199. Strain SAM85, which is a *lacI*^q^-containing version of BTH101, was made by conjugation of the bacterial two-hybrid host strain BTH101 (F^−^) with XL1-Blue (F^+^). This strain was used instead of BTH101, as it gave reduced background in our MacConkey plate assays. Strain JingY90 was constructed by introducing a *phoQ* deletion into JingY34 using P1 transduction, followed by kanamycin resistance cassette removal. Plasmids expressing *gfpA206K* fusions to *mgrB* mutants (pSY3-9, pSY72-75, pJC1-7, and pTG1-15) were created by either QuikChange site-directed mutagenesis (Stratagene) or inverse PCR (58), using pAL38 as a template. Plasmids expressing *gfpA206K* fusions of *mzrA* (pSY29), *safA* (pSY31), and *mgrB* (pSV1 to pSV8) orthologs were constructed by Gibson assembly (59).The gene fragments for *mzrA* and *safA* were PCR amplified from the MG1655 genome, and gene fragments corresponding to the different *mgrB* orthologs were synthesized commercially (Integrated DNA Technologies, Inc.). Plasmids pSY32 (MzrA TM swap) and pSY33 (SafA TM swap) were made by inverse PCR using pSY29 and pSY31 as templates, respectively. Plasmid pSY34 expressing T18–MgrB-24 only was created by inverse PCR using pAL33 as a template. For all the plasmids mentioned above, see Table S1C for the specific primer sequences used for each construction. Plasmids pSY61, pSY64, and pSY68 were constructed as follows: *atoS*, *cpxA*, and *phoR* were PCR amplified using the MG1655 genome as the template and the primer pairs atoS-xbaI-U1/atoS-kpnI-L1, cpxA-xbaI-U1/cpxA-kpnI-L1, and phoR-xbaI-U2/phoR-kpnI-L1, respectively; digested with XbaI/KpnI restriction enzymes; and then cloned into pKT25 at XbaI/KpnI sites. Plasmid pJY151 was constructed by inserting PCR-amplified *mgrB* into SacI/XbaI sites in the pBAD33RBS vector. Plasmid pJY569 expressing mCherry-MgrB was generated by inserting *mgrB* downstream of *mcherry* at BamHI/PstI sites in pBO2 (60). Plasmid pJY573 was generated by inserting *mneongreen* downstream of *phoQ* at SalI/HindIII sites in pBAD33RBS *phoQ* (14). Plasmids expressing *mgrB* variants were constructed using a Q5 site-directed mutagenesis kit (NEB). All the constructs were confirmed by DNA sequencing.

### Media and growth conditions.

Liquid cultures were grown at 37°C with aeration, unless otherwise indicated, in either Luria-Bertani (LB) Miller medium (Fisher Scientific) or minimal A medium (61) containing 0.2% glucose, 0.1% Casamino Acids, and 1 mM MgSO_4_. For routine growth on solid medium, LB or minimal medium containing bacteriological-grade agar (Fisher Scientific) was used. The antibiotics ampicillin, kanamycin, and chloramphenicol were used at concentrations of 50 to 100, 25 to 50, and 20 to 34 μg ml^−1^, respectively. The *lac* and *trc* promoters were induced with isopropyl-β-d-1-thiogalactopyranoside (IPTG) at a final concentration of 10 μM in FRET analysis or 1 mM when indicated. When IPTG is not mentioned in the description of the culture conditions, basal transcription from the *trc* promoter was used to drive expression. The *araBAD* promoter was induced with 0.008% arabinose. For bacterial two-hybrid screening, MacConkey-maltose indicator plates were prepared as described previously (36) using MacConkey agar base powder (Difco), 100 μg ml^−1^ ampicillin, 25 μg ml^−1^ kanamycin, 0.5 mM IPTG, and 1% maltose.

### β-Galactosidase reporter gene assay.

Cultures of strain AML67 carrying *gfpA206K* fusions of either wild-type *mgrB* (pAL38), *mgrB* mutants (pSY3-9, pSY72-75, pJC1-7, and pTG1-15), *mgrB* orthologs (pSV1-8), or *gfpA206K*-only (pAL39) plasmids were grown overnight in LB with 50 μg ml^−1^ ampicillin. The next day, they were diluted 1:1,000 in the same medium and grown to an optical density at 600 nm (OD_600_) of ∼0.3 to 0.4 at 37°C. The cells were permeabilized with chloroform-SDS and assayed as described previously (61). For high-throughput measurements in a 96-well format, the protocol was modified as described by Thibodeau et al. (62).

### GFP reporter assay.

An E. coli *imp-4213* strain carrying a GFP reporter plasmid (pUA66 P*_mgtLA_-gfp*) was grown overnight at 37°C in LB medium supplemented with 1 or 10 mM MgSO_4_ and then diluted 1:100 in fresh MgSO_4_-supplemented LB medium. DMSO, MgrB, or SafA C-terminal peptides (synthesized by GenScript) were added to the culture in the indicated amounts. The cultures were grown at 37°C with vigorous shaking for 2 h. The fluorescence of the cells was then monitored with a BD LSR Fortessa SORP flow cytometer (BD Biosciences), and the acquired data were analyzed as described previously (14). To test the functions of MgrB variants, the E. coli MG1655 Δ*mgrB* strain was transformed with a GFP reporter plasmid (pUA66 P*_mgtLA_-gfp*) (14) and a pBAD33 vector carrying a wild-type or mutant *mgrB* gene. The resulting transformants were grown overnight at 37°C in LB medium supplemented with 10 mM MgSO_4_ and then diluted 1:100 in fresh LB medium supplemented with 1 mM MgSO_4_ and 0.008% arabinose. The cultures were grown at 37°C with vigorous shaking for 2 h to reach early log phase (OD = 0.4 to 0.5). The fluorescence of the cells was monitored, and the acquired data were analyzed as described previously (14). The normalized MgrB mutant activity was calculated as follows: activity^mut^ = (fluorescence^no-MgrB control^ − fluorescence^MgrB mut^)/(fluorescence^no-MgrB control^ − fluorescence^MgrB WT^) × 100%. Where appropriate, 50 μg ml^−1^ kanamycin and 34 μg ml^−1^ chloramphenicol were added to the growth medium.

### Acceptor photobleaching FRET analysis.

The expression of PhoQ-mNeonGreen and mCherry-MgrB was induced in an E. coli Δ*phoQ* Δ*mgrB* strain with 0.008% arabinose and 10 μM IPTG, respectively. The cultures were grown to mid-log phase (OD = 0.6) in LB medium supplemented with 10 mM MgSO_4_ and appropriate antibiotics. Cells were harvested and washed two times with prechilled tethering buffer (20 mM potassium phosphate [pH 7.0], 1 μM methionine, 10 mM lactic acid, and 10 mM magnesium sulfate). The surface of a glass bottom 24-well plate (Greiner) was treated with 0.1% poly-l-lysine (Sigma) for 10 min at room temperature, followed by rinsing with tethering buffer. The cells were then added to the wells and incubated at room temperature for 10 min to allow attachment. Unattached cells were removed by washing two times, and attached cells were overlaid with tethering buffer afterward.

Acceptor photobleaching FRET was performed using a dual-layer Nikon Ti-E inverted fluorescence microscope equipped with a fluorescence lamp (X-cite Exacte; Lumen Dynamics), a perfect focus system (PFS), and NIS Elements AR software (version 4.40 Nikon). Images were acquired through a 40× Plan Apo 0.95-numerical-aperture (NA) objective in the mNeonGreen (donor) and mCherry (acceptor) channels; excitation power was adjusted both by controlling the fluorescence lamp output and with neutral-density (ND) filters. Specifically, the donor was excited at 482/18 nm and its emission was detected at 525/50 nm, and the acceptor was excited at 585/29 nm while its emission was detected at 647/57 nm. The excitation and emission filters were mounted on excitation and emission filter wheels, respectively, while beam splitters (495LP for mNeonGreen excitation and 605LP for mCherry excitation) were on the lower dichroic wheel of the microscope. Images were recorded using an iXon 897-X3 EM-charge-coupled-device (CCD) camera (Andor), the acquisition time was set to 1 s for both channels, and EM gain was kept in the range of 100 to 280. Acceptor (mCherry) photobleaching was induced with a 150-mW diode-pumped solid-state (DPSS) 593-nm laser (Acal BFi) that was reflected on the sample by a zt594 DCRB laser beam splitter (AHF) mounted on the upper dichroic wheel.

For each well, we acquired two or three sequences of images of isolated sample areas with the following protocol: (i) first, 2 images were taken in the acceptor channel; (ii) then, 60 images were taken in the donor channel, followed by (iii) 10 s of acceptor photobleaching (no image acquisition); afterward, (iv) 40 images were taken in the donor channel, and then (v) 2 images were taken in the acceptor channel. In order to avoid donor fluorescence recovery from interrupted illumination, the sample was continuously illuminated with a 482/18-nm excitation light, even during step iii. FRET efficiency was calculated as the donor signal increase divided by the total donor signal after acceptor photobleaching. In order to correct for the donor photobleaching present during steps ii to iv, we performed linear fitting (RStudio) of the donor fluorescence signal versus time for both pre- and postbleaching curves.

### Bacterial two-hybrid assays.

For the colorimetric spot assay, multiple clones from the transformation plate were inoculated in 3 ml of LB containing 100 μg ml^−1^ ampicillin and 25 μg ml^−1^ kanamycin. Several clones were picked in order to reduce heterogeneity (36). Cultures were grown overnight at 30°C with shaking. The next day, 3 μl of each culture was spotted on MacConkey-maltose indicator plates, and the plates were then incubated at 30°C. For each pair of plasmids, triplicate experiments were performed. Color change on the indicator plates was recorded after 48 h of incubation. Alternatively, β-Gal assays were performed on the overnight liquid cultures prepared as described for the colorimetric assay.

### Random mutagenesis of the PhoQ TM2-HAMP region.

A GeneMorph II random-mutagenesis kit (Stratagene) was used to mutagenize the TM2-HAMP-encoding region of *phoQ* (in pBAD33 *phoQ*) using primers that specifically target the region. The library was transformed into an E. coli Δ*phoQ* Δ*mgrB* strain carrying a plasmid GFP reporter (pUA66 P*_mgtLA_-gfp*) and an MgrB K2D K3D expression plasmid (pTrc99a *mgrB K2DK3D*). The transformants were grown on LB plates containing arabinose to induce PhoQ expression. E. coli colonies with repressed PhoQ have weak green fluorescence. Dim colonies were selected, and their plasmids were sequenced to identify mutations in PhoQ. Site-directed mutagenesis was performed using a Q5 site-directed mutagenesis kit (NEB).
